# *Xiaoyukang Jiaonang* Promotes the Degradation of Hypoxia-Inducible Factor 1*α* and Antiangiogenesis and Anti-Inflammation in Chronic Subdural Hematoma Rat Model

**DOI:** 10.1155/2020/2305017

**Published:** 2020-04-06

**Authors:** Jialin Liu, Xiaoke Dong, Zhonghao Li, Gesheng Wang, Yujia Zhou, Chongchong Liu, Kaiyue Wang, Lili Li, Jinmin Liu

**Affiliations:** ^1^Department of Neurosurgery, Dongfang Hospital Beijing University of Chinese Medicine, Beijing 100078, China; ^2^Department of Neurology, Dongfang Hospital Beijing University of Chinese Medicine, Beijing 100078, China

## Abstract

*Xiaoyukang Jiaonang* (XYK) is a Chinese patent medicine approved by the National Medical Product Administration which is used to treat intracranial hematoma in China. In this study, we observed the molecular mechanism of XYK in hypoxia-inducible factor 1*α* (HIF-1*α*), inflammation and angiogenesis of chronic subdural hematoma (CSDH). The CSDH model was made by using internal iliac vein blood of Wister rats, and rats were divided into sham group, CSDH group and XYK group. The rats in the XYK group were gavaged with *Xiaoyukang Jiaonang* (185 mg/kg) for 7 days, and rats in the CSDH group and sham group were gavaged with the same amount of physiological saline for 7 days. In the CSHD rat model, active inflammation and angiogenesis were observed around the hematoma. XYK promoted the ubiquitination and degradation of HIF-1*α*, and reduced the concentration of VEGF and the ratio of angiopoietin-1/angiopoietin-2. XYK reduced proinflammatory cytokines and increased anti-inflammatory cytokine. In tissue section, XYK reduced the size of the hematoma and membrane, and reduced vWF positive cells in membrane. Furthermore, the endothelial progenitor cells in blood decreased as well. Overall, XYK shows anti-inflammatory and antiangiogenesis effects which may relate to the degradation of HIF-1*α*.

## 1. Introduction

Chronic subdural hematoma (CSDH) is a common neurological disorder in elderly individuals, and its incidence is rising due to an ageing population and increasing use of anticoagulant and antiplatelet medications. Surgical evacuation via burr hole craniostomy is the most common treatment, but approximately 10–20% of surgically treated patients experience postoperative recurrence. Adjuvant treatments are needed in the management of CSDH, which may reduce both recurrence and the need for surgery [1]. Inflammation and angiogenesis are two important pathophysiological processes of CSDH [2]. Inflammation in CSDH appears to be mediated by a range of inflammatory cytokines. In both blood and hematoma fluid, inflammatory cytokines such as tumor necrosis factor (TNF)-*α*, interleukin (IL)-6 and IL-10 raise, and the anti-inflammatory activities in the hematoma may play a role in the risk of a recurrence of CSDH [3]. Vascular endothelial growth factor (VEGF) plays an important role in angiogenesis in patients with CSDH. The concentration of VEGF in subdural hematoma fluid is higher than in serum and is of great importance in the progression of CSDH [4]. Additionally, hypoxia-inducible factor-1 alpha (HIF-1*α*) can induce the release of VEGF [5].

*Xiaoyukang Jiaonang* (XYK) is a Chinese patent medicine approved to treat intracranial hematoma in China (GUOYAOZHUNZI Z20026074). The formula of XYK is made up with *Angelica sinensis* (Oliv.) Diles (*Danggui*, Angelicae Sinensis Radix), *Caesaplinia sappan L*. (*Sumu*, Sappan Lignum), *Aucklandia lappa* DC. (*Muxiang*, Aucklandiae Radix), *Paeonia lactiflora* Pall. (*Chishao*, Paeoniae Radix Rubra), *Lycopus lucidus* Turca. ex Benth. (*Zelan*, Lycopi Herba), *Boswellia carteri* Birdw (*Ruxiang*, Olibanum), *Rehmannia glutinosa* (Gaertn.) DC. (*Dihuang*, Rehmanniae Radix), *Alisma plantago-aquatica* subsp. *Orientale* (Sam.) Sam. (*Zexie*, Alismatis Rhizoma), *Commiphora myrrha* (Nees) Engl. (*Moyao*, Myrrha), *Ligusticum striatum* DC. (*Chuanxiong*, Chuanxiong Rhizoma), *Clematis armandii* Franch. (*Chuanmutong*, Clematidis Armandii Caulis), *Cyathula officinalis* K.C.Kuan (*Chuanniuxi*, Cyathulae Radix), *Prunus persica* (L.) Batsch (*Taoren*, Persicae Semen), *Dipsacus asper* Wall. ex C.B. Clarke (*Xuduan*, Dipsaci Radix), *Glycyrrhiza uralensis* Fisch. (*Gancao* Glycyrrhizae Radix et Rhizoma), *Carthamus tinctorius L*. (*Honghua* Carthami Flos), and *Cyperus rotundus L*. (*Xiangfu*, Cyperi Rhizoma). The efficacy of XYK in traditional Chinese medicine (TCM) is activating blood flow, removing blood stasis, alleviating swelling and relieving pain. In TCM clinical practice, XYK can be used for diseases such as CSDH [6], traumatic brain injury [7], hemorrhagic infarction [8] and intracerebral hemorrhage [9] which match with the pathogenesis of blood stasis. In cerebral ischemia-reperfusion injury rat model, XYK can reduce the serum TNF-*α* and IL-*β* contents, which imply XYK may have anti-inflammatory effect [10]. In a pilot clinical study, XYK improved the Barthel Index, activities of daily living and Glasgow Outcome Scale in CSDH patients with evacuation via burr hole craniostomy [6], but the pharmacology of XYK for CSDH is still unclear. In this study, we used a CSDH rat model to explore the role of XYK in HIF-1*α,* angiogenesis and inflammation.

## 2. Materials and Methods

### 2.1. Preparation of *Xiaoyukang Jiaonang*

The formula of XYK was motioned above. The preparation process conditions were confirmed as follows: water extraction volume of 6–7 times, extraction time of 2 h, ethanol concentration of 85% with the volume of 7–8 times, extraction time of 1 h twice, the closed placement time of 12 h, the optimum ethanol concentration of 95% and drying temperature of 70°C [11]. The XYK used in our study was purchased from Qinghai Ecion Pharmaceutical Co., Ltd.

### 2.2. Animals

All experiments were approved by local authorities and conducted in accordance with the Animal Research: Reporting of In Vivo Experiments guidelines. Fourteen male Wistar rats bought from Qinglong Mountain Animal Breeding Farm (License number: SCXK-2017-0001, weight: 250–300 g) were raised in a temperature-controlled room with 12-h light-dark cycle and free chow and water at Beijing PoolingMed Co., Ltd. Rats were assigned to 3 groups by random number table: sham group (*n* = 4), CSDH group (*n* = 4) and XYK group (*n* = 6).

### 2.3. CSDH Model

The subdural hematoma model was induced by a surgery published before [12]. Briefly, rats were anesthetized intraperitoneally with 1% pentobarbital solution (40 mg/kg) and positioned in a stereotaxic frame (ALC-H, Shanghai Alcott Biotech Co., Ltd.). A small sphenoid burr hole (0.9 mm in diameter) was performed by using a wedge bit on the left coronal suture whose lateral distance to the sagittal suture was 3 mm. Then, endocranium of the rat was scuffed with a small hooked needle (with a diameter of 0.3 mm) under the microscope. Then, 300 *μ*l autologous venous blood collected from angular vein of the rat was injected into its subdural space at a rate of 50 ml/min by using a 20-gauge Venflon catheter with a tapered tip. Rats in the sham group suffered the same procedures without blood injection. The rats in the XYK group were gavaged with XYK (185 mg/kg), and rats in the CSDH group and sham group were gavaged with the same amount of physiological saline. Seven days after surgery, rats were anesthetized using pentobarbital solution, and 1 ml venous blood was sampled from inner canthus. Then rats were euthanized by decapitation method for histological and histochemical brain tissue analysis.

### 2.4. Hematoxylin and Eosin (HE) Staining

The tissue was fixed in 4% paraformaldehyde for 24 hours and then processed with dehydration clearing and wax embedding. 4 *μ*m slices were dewaxed to water, then stained with hematoxylin dye solution (Sigma, H9627) and eosin staining (Sinopharm, 71014544) in order, and observed and photographed under a microscope (OLYMPUS, BX53).

### 2.5. Immunohistochemistry

The tissue was fixed in 4% paraformaldehyde for 24 hours and then processed with dehydration clearing and wax embedding. 4 *μ*m slices were dewaxed to water and heated with electric stove for antigen repair. Goat serum (Solarbio, SL038) was used to reduce nonspecific staining. Primary antibody von Willebrand factor (vWF, Cloud-Clone, PAA833Ra01, 1 : 50) was added and slices were incubated in a wet box at 4°C for a night. Then HRP labeled Goat anti-Rabbit secondary antibody (Beyotime Biotechnology, A0277, 1 : 100) was added. DAB Color-substrate solution (Agilent Technologies, K5007) was used. Harris hematoxylin (Sigma, H9627) was used for staining. Slices were observed and collected as images under the microscope (OLYMPUS, BX53).

### 2.6. ELISA

Hematoma membrane was ground at a low temperature and diluted with 0.01 mol/L PBS and then put in a centrifuge with 5000 rpm for 5 minutes, and the supernatant was separated and collected. For cytokine assay in the hematoma membrane, TNF-*α* (Cloud-Clone, SEA133Ra), IL-6 (Cloud-Clone, SEA079Ra) and IL-10 (Cloud-Clone, SEA056Ra) were measured by ELISA kits according to the manufacturer's instructions.

### 2.7. Western Blot Analysis

Hematoma membrane was ground at a low temperature and quantified with BCA protein assay (Beyotime Biotechnology, P0010). Equal amounts of total proteins were subjected to 10% (W/V) SDS-PAGE and transferred onto nitrocellulose membranes (Millipore, ISEQ15150). Membranes were then blocked with 5% skim milk for 1 hour at room temperature and probed with anti-angiopoietin-1 (anti-Ang-1, Affinity, AF5184, 1 : 1000), anti-angiopoietin-2 (anti-Ang-2, Affinity, AF5124, 1 : 1000), anti-VEGF (Cloud-Clone, PAA143Ra01, 1 : 500), anti-E3 ubiquitin-protein ligase parkin (affinity, AF0235, 1 : 1000), anti-26S proteasome (Santa Cruz Biotechnology, sc-73488, 1 : 500), anti-HIF-1*α* (Cloud-Clone, PAA798Ra01, 1 : 500) and anti-GAPDH (Goodhere Biological Technology Group, AB-P-R001, 1 : 1000) antibodies at 4°C overnight. Subsequently, membrane was incubated with horseradish peroxidase-conjugated goat anti-rabbit or rabbit anti-goat IgG for 2 hours at 37°C, after 5–6 times TBST washing, and then reacted with an enhanced ECL substrate. The result of chemiluminescence was recorded with an imaging system and semiquantified using the Image *J* software (NIH Image, Bethesda, Maryland, USA).

### 2.8. Flow Cytometry

1ml venous blood from inner canthus was used to extract nucleated cells. And CD133 (Novus, NB120-16518G, 1 : 100) and CD34 (Novus, NBP2-47911PE, 1 : 100) antibodies were used to label endothelial progenitor cells (EPCs). After incubation, centrifugation, washing, and filtration were conducted through microgrid and then flow cytometry detection was conducted after labeling.

### 2.9. Statistical Analysis

All data were obtained in form of mean ± standard deviation, and data in different groups were analyzed by one-way analysis of variance and post hoc Scheffe tests. Two-tailed *P* < 0.05 is considered as statistically significant. Graphpad Prism 6.0 software (GraphPad Software, Inc., San Diego, CA, USA) was used for statistical analysis and visualization.

## 3. Results

Overall animals' health evaluation such as average weight variation, skin ulceration, loss of activity, diarrhea, hematuria, salivation, tremor, seizure, vomiting, edema and aggressive behavior was observed in all control and test groups before and during the total period of experiment. Three rats in the XYK group and 1 rat in the CSDH group died after the surgery. Hence, there were 10 rats (4 in sham group, 3 in CSDH group and 3 in XYK group) in the analysis.

### 3.1. HIF-1*α* Ubiquitination and Angiogenesis-Related Proteins Decreased in the XYK Group

**Figure 1:**
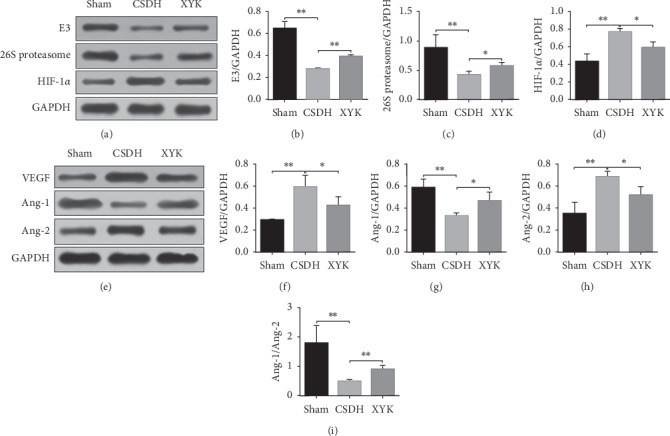
In the XYK group, HIF-1*α* and VEGF decreased, E3 ubiquitin-protein ligase parkin and 26S proteasome protein increased, and the Ang-1/Ang-2 ratio increased in the hematoma. Western blots analysis of HIF-1*α*, E3 ubiquitin-protein ligase parkin, and 26S proteasome protein in hematoma (A-D). Western blots analysis of VEGF, Ang-1, and Ang-2 (E-I). ^*∗*^*P* < 0.05 and ^*∗∗*^*P* < 0.01. Ang, angiopoietins; CSDH, chronic subdural hematoma HIF, hypoxia-inducible factor; VEGF, vascular endothelial growth factor; XYK, *Xiaoyukang Jiaonang*.

In Western blot analysis, compared with the sham group, HIF-1*α*, VEGF and Ang-2 in the hematoma of the CSDH group increased significantly, E3 ubiquitin-protein ligase parkin, 26S proteasome and Ang-1 in the hematoma of the CSDH group decreased significantly, and the Ang-1/Ang-2 ratio decreased significantly. In the XYK group, HIF-1*α* and VEGF decreased significantly, E3 ubiquitin-protein ligase parkin and 26S proteasome protein in the hematoma increased significantly, and the Ang-1/Ang-2 ratio increased significantly compared with the CSDH group (Figure 1).

### 3.2. TNF-*α* and IL-6 Decreased and IL-10 Increased in the XYK Group

**Figure 2:**
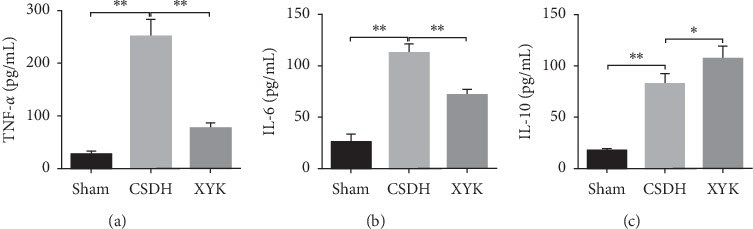
TNF-α and IL-6 decreased while IL-10 increased in the XYK group. ELISA results of TNF-*α* (a), IL-6 (b), and IL-10 (c) in hematoma. Data are mean ± SD. Analyzed by one-way analysis of variance. ^*∗*^*P* < 0.05 and ^*∗∗*^*P* < 0.01. CSDH, chronic subdural hematoma; IL, interleukin; TNF, tumor necrosis factor; XYK, *Xiaoyukang* Jiaonang.

Compared with the sham group, TNF-*α*, IL-6, and IL-10 in hematoma increased significantly in the CSDH group (TNF-*α*: 26.51 ± 5.35 vs 250.31 ± 26.99 pg/ml, *P* < 0.01; IL-6: 25.76 ± 6.39 vs 112.77 ± 6.91 pg/ml, *P* < 0.01; IL-10 : 17.60 ± 1.53 vs 82.76 ± 8.12 pg/ml, *P* < 0.01.  Figure 2). In the XYK group, TNF-*α* and IL-6 decreased while IL-10 increased compared with the CSDH group (TNF-*α*: 76.48 ± 8.47 vs 250.31 ± 26.99 pg/ml, *P* < 0.01; IL-6: 71.51 ± 4.68 vs 112.77 ± 6.91 pg/ml, *P* < 0.01; IL-10 : 107.37 ± 9.63 vs 82.76 ± 8.12 pg/ml, *P* < 0.05; Figure 2).

### 3.3. Hematoma Membrane vWF Expression Decreased in the XYK Group

**Figure 3:**
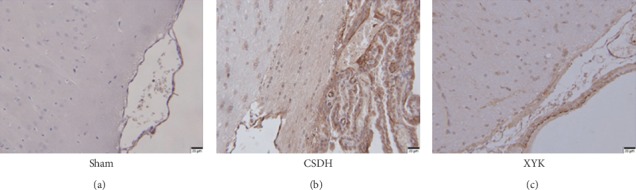
Hematoma membrane vWF expression decreased in the XYK group. Scale bars 20 *μ*m. CSDH, chronic subdural hematoma; XYK, *Xiaoyukang Jiaonang*; vWF, von Willebrand factor.

The expression of vWF increased significantly in the CSDH model group, indicating abundant angiogenesis; after treated with Xiaoyukang Jiaonang, the expression of vWF decreased, indicating reduced angiogenesis. No hematoma was found in the sham group, and there was no positive expression of vWF in the sham group (Figure 3).

### 3.4. Hematoma Membrane in the XYK Group Was Thicker

**Figure 4:**
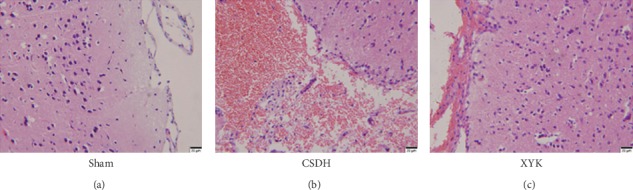
The hematoma and membranous tissue were decreased in the XYK group compared with the CSDH group. Scale bars 20 *μ*m. CSDH, chronic subdural hematoma; XYK, *Xiaoyukang Jiaonang.*

Obvious hematoma and membranous tissue at the edge of hematoma was found in the CSDH model group, with thin and incomplete membrane tissue, and inflammatory cells were visible. In the XYK group, the hematoma decreased, the membranous tissue was thickened, and inflammatory cells were increased. No hematoma was found in the sham group (Figure 4).

### 3.5. Percent of EPCs in Peripheral Blood Decreased in the XYK Group

**Figure 5:**
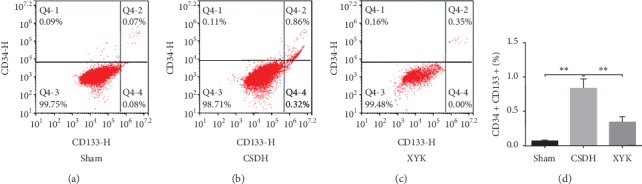
Percent of EPCs (CD133 + CD34 +) reduced in the XYK group detected by flow cytometry. Data are mean ± SD analyzed by one-way analysis of variance. ^*∗∗*^*P* < 0.01. CSDH, chronic subdural hematoma; EPC, endothelial progenitor cell; XYK, *Xiaoyukang Jiaonang*.

Compared with the sham group, the percent of EPCs (CD133 + CD34 +) increased significantly in the CSDH group (0.07 ± 0.01 vs 0.84 ± 0.11%, *P* < 0.01.  Figure 5). In the XYK group, the percent of EPCs (CD133 + CD34 +) reduced significantly (0.34 ± 0.07 vs 0.84 ± 0.11%, *P* < 0.01. Figure 5).

## 4. Discussion

In our study, the rat model of CSDH showed large hematoma and membrane, and the inflammation and angiogenesis were active. In the XYK group, HIF-1*α* reduced and the E3 ubiquitin-protein ligase parkin and 26S proteasome which related to the ubiquitination and degradation of HIF-1*α* were activated and showed antiangiogenesis effects (reduced vWF positive cells in membrane and ECPs in blood; decreased VEGF and Ang-2; increased Ang-1 and the ratio of Ang-1/Ang-2) and anti-inflammatory (reduced TNF-*α* and IL-6, but increased IL-10). Overall, the size of hematoma and membrane reduced.

HIF-1α is a ubiquitous transcription factor in cells of human and mammals, and it can express under nonhypoxic conditions but can be made for proteasomal degradation by ubiquitin ligase complex due to the two specific acetylation of two lysine residues on HIF-1*α* protein which allows for the binding of von Hippel-Lindau protein that interacts with the elongin C protein [13]. Parkin is a RANG finger-containing protein, which can function as an E3 ubiquitin ligase to ubiquitinate and degrade substrate proteins and has been reported to play an important role in regulating mitochondrial homeostasis, antioxidative stress, and mitophagy. Recently, a study showed parkin expression is inversely correlated with HIF-1α expression in breast cancer, and parkin could interact with HIF-1*α* and promote HIF-1α degradation through ubiquitination [14]. 26S proteasomal degradation system is involved in the degradation of HIF-1*α* after ubiquitination. Inhibiting 26S proteasome can increase the HIF-1*α* and VEGF protein level [15]. HIF-1*α* is a mediator of cellular response to hypoxia condition which can induce VEGF gene transcription by binding to the hypoxia response element in the VEGF promoter region. In the hematoma of CSDH patients, VEGF expression was significantly correlated to HIF-1*α* expression and VEGF concentration in the hematoma [5]. In the CSDH rat, VEGF and HIF-1*α* were significantly increased which implied the active angiogenesis. Nevertheless, XYK gavage could reduce the level of VEGF and HIF-1*α*; besides, the level of parkin and 26S proteasome increased, which implied XYK can promote the degradation of HIF-1*α*. The degradation of HIF-1α may contribute to reducing of VEGF.

Angiogenesis is an important pathology in the formation of CSDH. Angiopoietins and VEGF are two families of specific endothelial growth factors that play important roles during the formation, growth, and maturation of new vessels [2]. VEGF has a proangiogenic and potentially proinflammatory role. In patients with CSDH, high VEGF concentration in the hematoma fluid is of major pathophysiological importance in the generation and steady increase of the hematoma volume, which is essential for the formation and remodeling of new vessels [4]. VEGF and the proangiogenic factor angiopoietin 2 create an unstable condition with the continuous formation of new and immature capillaries causing extravasation and recurrent microbleeds [16]. Ang-1 can stabilize newly formed blood vessels after pathological insults in mice and stabilize the cortical actin cytoskeleton and promote cell junction integrity and decrease expression of Ang-2 and inflammatory genes in cultured endothelial cells. And Ang-2 can inhibit the function of Ang-1. In inflammation, decreased Ang-1/Ang-2 ratio may contribute to enhanced vessel permeability and vascular destabilization [17]. In our study, VEGF decreased and the ratio of Ang-1/Ang-2 increased which implied XYK has the antiangiogenesis effect and promotes the stabilization of new born vessels in CSDH.

Inflammation is also an important pathology in the formation of CSDH. Many inflammatory cells are involved in the CSDH, such as neutrophils, lymphocytes, macrophages, and eosinophils [18], and both pro- and anti-inflammatory cytokines are raised in CSDH, but proinflammatory cytokines are more than anti-inflammatory cytokines [19]. Our study showed in CSDH rat model both proinflammatory cytokines TNF-*α* and IL-6 and anti-inflammatory cytokine IL-10 increased. In the XYK group, TNF-*α* and IL-6 significantly decreased, but IL-10 significantly increased, which implied XYK has anti-inflammatory effect in the balance of pro- and anti-inflammatory cytokines.

EPCs originate from either bone marrow or other tissues (e.g., spleen, vessel wall, adipose tissue, placenta, etc.), which can contribute to replacing the injured endothelium and release a number of proangiogenic molecules [20]. In CSDH patients, the level of EPCs is lower than that in healthy volunteers and is much lower in patients with recurrent CSDH [21]. But in our study, the level of EPCs was increased in CSDH rat model, and XYK could decrease the high level of EPCs, which may relate to the inhibition of inflammation and angiogenesis.

In the long history of Chinese clinical practice, TCM has formed a unique perspective of pathogenesis and treatment for diseases. The pathogenesis of CSDH in TCM is qi stagnation and blood stasis which may be caused by head trauma. In accordance with this pathogenesis, herbs and formulas that could activate qi, activate blood, and resolve stasis are used to treat CSDH in TCM which can achieve a good efficacy such as XYK [22]. However, it is always difficult to discover the pharmacology of herbs and formulas by modern technologies. There may be dozens of herbs in one herbal formula; for example, there are seventeen herbs in XYK, which may contain hundreds and thousands of compounds and each compound may modulate multiple proteins, just like XYK can regulate many proteins in inflammation and angiogenesis of CSDH. What's more, there are also thousands of targets in the treatment of diseases. The relationship between herbs and diseases is complex. Nowadays, with advances in systems biology, a new paradigm in drug discovery integrating of network biology and polypharmacology was created: network pharmacology [23]. Network pharmacology in TCM also named TCM network pharmacology can be used as a new research paradigm for translating TCM from an experience-based medicine to an evidence-based medicine system, accelerating TCM drug discovery, and also improving current drug discovery strategies [24].

In clinical practice, several nonsurgical treatments, such as dexamethasone, cholesterol-lowering drug, and angiotensin-converting enzyme inhibitors which target the inflammation and angiogenesis, are used to treat CSDH [16]. Cholesterol-lowering drug atorvastatin can decrease the TNF-*α* and IL-6 level as well as VEGF gene expression in rat model [25], and clinical trial also showed atorvastatin may be a safe and efficacious nonsurgical alternative for treating patients with CSDH [26]. Hence, XYK has the effect of antiangiogenesis and anti-inflammation and has the potential to treat CSDH. Further clinical studies are needed to confirm these results.

## 5. Conclusions

In conclusion, XYK can reduce the size of the hematoma and membrane in CSDH rat model and shows anti-inflammatory and antiangiogenesis effects. The degradation of hypoxia-inducible factor 1α is activated as well which may contribute to those effects. Further studies are needed to confirm these results.

## Data Availability

The datasets used and/or analyzed during the current study are available from the corresponding author on reasonable request.
